# Acamprosate Is a Substrate of the Human Organic Anion Transporter (OAT) 1 without OAT3 Inhibitory Properties: Implications for Renal Acamprosate Secretion and Drug–Drug Interactions

**DOI:** 10.3390/pharmaceutics12040390

**Published:** 2020-04-24

**Authors:** Irina E. Antonescu, Maria Karlgren, Maria L. Pedersen, Ivailo Simoff, Christel A. S. Bergström, Sibylle Neuhoff, Per Artursson, Bente Steffansen, Carsten Uhd Nielsen

**Affiliations:** 1 Department of Physics, Chemistry and Pharmacy, University of Southern Denmark, Campusvej 55, 5230 Odense, Denmark; antonescu@sdu.dk (I.E.A.); mariap@sdu.dk (M.L.P.); 2 Department of Pharmacy, Uppsala University, Husargatan 3 BMC, SE-751 23 Uppsala, Sweden; maria.karlgren@farmaci.uu.se (M.K.); Christel.Bergstrom@farmaci.uu.se (C.A.S.B.); Per.Artursson@farmaci.uu.se (P.A.); 3 Uppsala University Drug Optimization and Pharmaceutical Profiling Platform (UDOPP), Department of Pharmacy, Uppsala University, Husargatan 3 BMC, SE-751 23 Uppsala, Sweden; ivailo.simoff@farmaci.uu.se; 4 Certara UK Limited, Simcyp Division, Level 2-Acero, 1 Concourse Way, Sheffield S1 2BJ, UK; sibylle.neuhoff@certara.com; 5 LEO Pharma, Industriparken 55, 2750 Ballerup, Denmark; FFBDK@leo-pharma.com

**Keywords:** acamprosate, probenecid, organic anion transporter, OAT1, OAT3, HEK293 cells, renal carrier, secretion, drug–drug interaction

## Abstract

Acamprosate is an anionic drug substance widely used in treating symptoms of alcohol withdrawal. It was recently shown that oral acamprosate absorption is likely due to paracellular transport. In contrast, little is known about the eliminating mechanism clearing acamprosate from the blood in the kidneys, despite the fact that studies have shown renal secretion of acamprosate. The hypothesis of the present study was therefore that renal organic anion transporters (OATs) facilitate the renal excretion of acamprosate in humans. The aim of the present study was to establish and apply OAT1 (gene product of *SLC22A6*) and OAT3 (gene product of *SLC22A8*) expressing cell lines to investigate whether acamprosate is a substrate or inhibitor of OAT1 and/or OAT3. The studies were performed in HEK293-Flp-In cells stably transfected with *SLC22A6* or *SLC22A8*. Protein and functional data showed that the established cell lines are useful for studying OAT1- and OAT3-mediated transport in bi-laboratory studies. Acamprosate inhibited OAT1-mediated *p*-aminohippuric acid (PAH) uptake but did not inhibit substrate uptake via OAT3 expressing cells, neither when applied concomitantly nor after a 3 h preincubation with acamprosate. The uptake of PAH via OAT1 was inhibited in a competitive manner by acamprosate and cellular uptake studies showed that acamprosate is a substrate for OAT1 with a K_m_-value of approximately 700 µM. Probenecid inhibited OAT1-mediated acamprosate uptake with a K_i_-value of approximately 13 µM, which may translate into an estimated clinically significant DDI index. In conclusion, acamprosate was identified as a substrate of OAT1 but not OAT3.

## 1. Introduction

**Figure 1:**
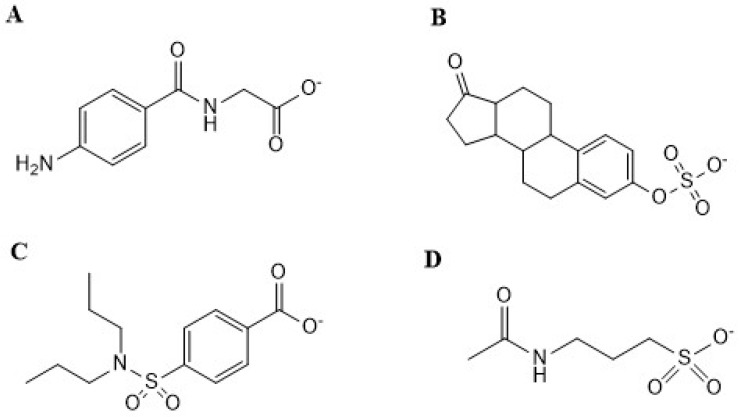
Chemical structures of (**A**) *p*-aminohippuric acid; (**B**) estrone-3-sulfate; (**C**) probenecid; (**D**) acamprosate (acetylhomotaurinate).

Acamprosate is a drug substance widely used in treating the symptoms of alcohol withdrawal and usually administered as enteric-coated tablets in doses of up to 2 g/day [1]. When administered orally or intravenously (bolus), acamprosate reaches unbound steady-state (C_max,u_) and initial (C_0,u_) plasma concentrations of 2–5 and 154–768 µM [2], respectively, for doses of 333–2130 mg. Acamprosate is a highly hydrophilic compound [3] (Figure 1), which carries a net negative charge at physiological pH of 7.4. Acamprosate has negligible plasma protein binding (f_u_ = 1) and is not metabolized in humans [4,5,6], yet its elimination mechanism(s) is not fully understood. It is known that renal clearance (CL_R_) accounts for >95% of the eliminated acamprosate dose [5,7] and pharmacokinetic studies suggest that an active acamprosate renal secretion occurs [8], since the observed renal clearance (CL_R_) of unbound acamprosate, 13.4–23.4 L/h [6,9,10,11], exceeds the glomerular filtration rate (GFR·f_u_) of 5.4–7.5 L/h in healthy adults. Thus, an active secretion component was estimated to contribute to more than 44% of the total acamprosate CL_R._ Moreover, after intravenous administration of 9.3 mg/kg acamprosate to rats together with 33.3 or 66.6 mg/kg probenecid, a decrease in the renal clearance of acamprosate was observed. The decrease occurred in a dose-dependent manner, from 0.35 L/h—a CL_R_ value almost close to the kidney blood flow in rats (0.55 L/h)—to 59% and 45% of the initial CL_R_, respectively [12]. Probenecid is a known inhibitor of several solute carriers, e.g., the organic anion transporter (OAT) 1 (SLC22A6; gene name *SLC22A6*) and OAT3 (SLC22A8; gene name *SLC22A8*) [13] expressed in the abluminal membrane of renal epithelial cells in the proximal tubule [14] in both humans [15] and rats [16,17]. Therefore, a competition between probenecid and acamprosate at these carriers could result in a decreased secretion and decreased clearance from the blood to the renal epithelial cells. However, no carriers of acamprosate have to the best of our knowledge been identified so far.

In a recent study in Caco-2 cells [18], preliminary data suggested that acamprosate did not inhibit substrate transport via PEPT1, TAUT, PAT1, EAAT1, B^0,+^AT/rBAT, OATP2B1, nor ASBT solute carriers (SLCs), while both experimental and modelling data suggested the paracellular pathway to be the main transport route for oral absorption. Still, acamprosate is secreted in the kidney, raising the question as to which SLCs are involved in this secretion. Since acamprosate and probenecid are both anionic compounds, investigating whether acamprosate interacts with organic anion transporters as either an inhibitor or substrate was chosen as a starting point for this investigation. The aim of the present study was therefore to establish OAT1- and OAT3-expressing cell lines to allow for investigating whether acamprosate is a substrate and/or inhibitor of OAT1 and/or OAT3.

HEK293-Flp-In cells stably transfected with *SLC22A6* or *SLC22A8* were characterized in two different laboratories, and in these models acamprosate demonstrated the ability to inhibit transport via OAT1, whereas no significant inhibition was observed for transport via OAT3. Moreover, probenecid inhibits the OAT1-mediated uptake of acamprosate at potentially clinically relevant concentrations.

## 2. Materials and Methods

The studies in the present work were conducted at two different research facilities. Some studies were conducted at Department of Pharmacy, Uppsala University (termed Lab. 1 throughout the paper) while some studies were conducted at the Department of Physics, Chemistry and Pharmacy, University of Southern Denmark (termed Lab. 2 throughout the paper).

### 2.1. Materials

Cell culture plasticware were from Corning Life Sciences and purchased through Sigma (Copenhagen, Denmark). CellBIND cell culture plates were from Corning Life Sciences (Amsterdam, The Netherlands). Luma plates were from PerkinElmer (Downers Grove, IL, USA). Ultima Gold scintillation liquid, *p*-[^14^C]-aminohippuric acid (52.7 mCi/mmol) and [^3^H]-estrone-3-sulfate (51.8 Ci/mmol) were from PerkinElmer (Skovlunde, Denmark). The isotopes had a radiochemical purity of >97%. Acamprosate calcium (purity >98%) was from Santa Cruz Biotechnology (Heidelberg, Germany). *P*-aminohippuric acid (purity >99%), estrone-3-sulfate (contains ≈35% Tris as a stabilizer; purity >98%), probenecid (purity >98%), NP-40 tergitol 70%, Dulbecco’s Phosphate buffer saline, sodium bicarbonate 7.5% and 4-(2-hydroxyethyl)-1-piperazineethanesulfonic acid (HEPES), Dulbecco’s modified Eagle medium (DMEM), L-glutamine (L-Glu, 200 mM), Fetal Bovine Serum (FBS), Triton X-100 10%, trypsin-EDTA (10x), Tris-HCl, Sucrose, EDTA, EGTA, and sodium dodecyl sulfate (SDS) were purchased from Sigma (Copenhagen, Denmark). The BCA protein assay kit and Hank’s balanced salt solution (HBSS) 10x were obtained from Thermo Fisher Scientific (Hvidovre, Denmark). Complete Protease inhibitor cocktail tablets were from Roche (Hvidovre, Denmark). The CellTiter-Glo Luminescent cell viability assay was from Promega (Madison, WI, USA). The water used in all the assays was from a Milli-Q water purification system with 0.22 µM Millipak filter (EMD Millipore, Temecula, CA, USA).

### 2.2. Establishment of Stable Cell Lines 

The *SLC22A6* open reading frame was amplified from a sequence-verified *SLC22A6* cDNA clone (Thermo Fischer Scientific Biosciences GmbH, St. Leon-Rot, Germany) using Platinum *Pfx* DNA polymerase (Thermo Fischer Scientific, Waltham, MA, USA) and the gene-specific primer pair 5′-GATATGGCCTTTAATGACCTCCTGCAG-3′/5′-GTCAGAGTCCATTCTTCTCTTGTGCTGAG-3′ (start and stop codons are underlined). For amplification of the *SLC22A8* open reading frame total human kidney RNA (AH diagnostics A/S, Aarhus, Denmark) was reverse transcribed using the Superscript III reverse transcriptase (Thermo Fischer Scientific, Waltham, MA, USA) according to the manufacturer’s instructions, followed by PCR amplification using Platinum *Pfx* DNA polymerase (Thermo Fischer Scientific, Waltham, MA, USA) and the gene-specific primer pair 5′-GATATGACCTTCTCGGAGATCCTGGAC-3′/5′-GTCAGCTGGAGCCCAGGCC-3′ (start and stop codons are underlined). The resulting PCR products were cloned into the TOPO expression vector pcDNA5/FRT/V5-His-TOPO (Thermo Fischer Scientific, Waltham, MA, USA). The inserted sequences were verified by DNA sequencing analysis (Uppsala Genome Center, Uppsala, Sweden). HEK293-Flp-In cells were co-transfected with the pOG44 vector (Thermo Fischer Scientific, Waltham, MA, USA) and either the constructed *SLC22A6*-pcDNA5/FRT/V5-His-TOPO, *SLC22A8*-pcDNA5/FRT/V5-His-TOPO expression vectors, or with empty pcDNA5/FRT/V5-His-TOPO and stable clones from the three cell lines were selected as previously described in Karlgren et al. [19]. The establishment of HEK293-Flp-In expressing OAT1, OAT3, and mock cell lines was done at Lab. 1.

### 2.3. LC/MS-MS-Based Protein Quantification

For protein quantification, 3 × 10^6^ HEK293-Flp-In OAT1, OAT3, or mock cells were lysed in 100 mM Tris-HCl-buffer, pH 7.4, containing 2% SDS and 50 mM DTT. Proteins were denatured at 95 °C. Samples were prepared for proteomic analysis using the multi-enzyme digestion filter-aided sample preparation (MED-FASP) protocol, where proteins were digested with Lys-C and trypsin [20]. Protein and peptide amounts were determined based on tryptophan fluorescence [21]. Peptides were separated on a reverse-phase EASY-spray LC column (50 cm × 75 µm inner diameter; Thermo Fisher Scientific, Waltham, MA, USA) packed with 2 µm C18 particles, using a 125-min acetonitrile gradient in 0.1% formic acid at a flow rate of 330 nL∙min^−1^. The LC was coupled to a Q Exactive HF mass spectrometer (Thermo Fisher Scientific, Waltham, MA, USA), operating in a data dependent TopN mode with survey scans at a resolution of 120,000, AGC target of 3 × 10^6^ and maximum injection time of 20 ms. The top 15 most abundant isotope patterns were selected from the survey scan with an isolation window of 1.4 *m/z* and fragmented with nCE at 28.5. The MS/MS analysis was performed with a resolution of 30,000, AGC target of 1 × 10^5^ and maximum injection time of 90 ms. The resulting MS data were processed with MaxQuant [22] where proteins were identified by searching MS and MS/MS data of peptides against the human UniProtKB. Protein concentrations were calculated using the Total Protein Approach [23]. Only proteins identified by at least three peptides were considered as quantified. The LC/MS-MS-based protein quantification was done at Lab. 1.

### 2.4. Cell Cultivation

Stably transfected HEK293-Flp-In expressing OAT1 and OAT3 and mock cells were grown in DMEM media containing FBS 10%, L-Glu 1%, and hygromycin 75 µg∙mL^−1^. For experiments, cells were seeded in DMEM media containing FBS 10% and L-Glu 1% either without (at Lab. 1) or with (Lab. 2) Phenol Red into 24-well or 96-well CellBIND plates at Lab. 1 or tissue culture treated 24-well or 96-well plates at Lab. 2, at a density of 6 × 10^5^ cells/well (3.15 × 10^5^ cells∙cm^−2^) or 1 × 10^5^ cells∙well^−1^ (3.12 × 10^5^ cells∙cm^−2^), respectively. The cells were incubated at 37 °C in a humidified atmosphere of 5% CO_2_/95% air and were used for uptake experiments 71–75 h after seeding in Lab. 1 or 30–48 h after seeding (Lab. 2). Preliminary uptake studies with established substrates showed no major differences in uptake for the post-seeding time interval. The stably transfected cell lines could both be propagated with a maintained function for at least 10 cell passages.

### 2.5. Buffers and Applied Solutions

Uptake studies were performed in HBSS buffered with 10 mM HEPES and the pH was adjusted to 7.40 ± 0.01 (at room temperature) with 1M NaOH to obtain the final buffer solution. Solutions containing the investigated radiolabeled and/or non-labelled compounds represent the donor solutions. All acamprosate concentrations are expressed as acetyl-homotaurine and not as the calcium salt. The donor solutions were prepared in the uptake buffer and had the pH adjusted as above. The osmolality of all applied solutions was 0.29–0.31 osmol/kg at room temperature.

### 2.6. Uptake Studies in HEK293-Flp-In OAT1, OAT3, and Mock Cells

All uptake experiments were performed on 24- or 96-well plates. Before the experiment initiation, the cells were incubated with either 500 or 200 µL∙well^−1^ prewarmed buffer for 10 min at 37 °C in a microplate incubator (VWR International AB, Stockholm, Sweden), without shaking. In the case of time-dependent uptake assay, the experiment was initiated by replacing the incubation buffer with 300 or 100 µL∙well^−1^ prewarmed donor solution containing 0.5 µCi∙mL^−1^ (9.5 µM) [^14^C]-PAH or 1 µCi/mL (0.02 µM) [^3^H]-E3S and placing the plate in the incubator at 37 °C and 100 rpm. The experiments were stopped after 1, 3, 5, 7.5, 10, and 20 min (Lab. 1), 1, 2, 3, 5, 10, and 15 min (Lab. 2) for [^14^C]-PAH uptake or 2.5, 5, 10, 15, 20, 25, 30 min for [^3^H]-E3S uptake (Lab. 2) by removing the applied solutions and washing immediately with 3 × 200 µL∙well^−1^ ice-cold buffer. For the dose-dependent OAT1 affinity study, the donor solution contained 0.02–9.5 µM [^14^C]-PAH supplemented with non-labelled PAH up to a total PAH concentration of 0.02–677 µM. For the dose-dependent OAT3 affinity study, the donor solution contained 0.02 µM [^4^H]-E3S supplemented with non-labelled E3S up to a total E3S concentration of 0.02–200 µM. For the inhibition studies, the donor solutions contained 9.5 µM [^14^C]-PAH or 0.02 µM [^3^H]-E3S and non-labelled probenecid (0–1022 µM) or acamprosate (0–33 mM). The experiments were stopped after 3 min (Lab. 2) or 5 min (Lab. 1) for [^14^C]-PAH or 20 min for [^3^H]-E3S, time points at which the substrate uptake was within the linear range. The experiment was stopped by adding 200 µL∙well^−1^ of ice-cold buffer and subsequent rinse three times with the same amount of buffer. In Lab. 1 the sample analysis of the OAT1/OAT3/mock studies consisted in removing the rinsing buffer and lysing the cells with 70 µL 1M NaOH, following by incubation for 60 min at 37 °C and 100 rpm. A total of 50 µL of cell lysate from each well was transferred to 96-well Luma plates bottom coated with dry scintillant. Then, 2∙20 µL of each donor and three 50 µL blank samples were applied on the same plate. The dry plates were covered with transparent seals and analyzed using a TopCount NXT Microplate Scintillation and Luminescence Counter (Perkin Elmer, Waltham, MA, USA). The remaining 20 µL cell lysate was neutralized with 20 µL 1M HCl∙well^−1^, samples were mixed thoroughly and the total protein content in all the wells was measured using a BCA protein assay kit, microplate method, and a Tecan Safire II fluorometer (Tecan Austria GmbH, Grödig, Austria). For the experiments in Lab. 2, the cells were lysed by adding 200 µL 0.1% Triton X-100 and incubated for 5 min at 37 °C and 100 rpm. Cell lysates were transferred to scintillation tubes together with 2 mL Ultima Gold scintillation liquid, mixed thoroughly and quantified by scintillation counting with a TriCarb 4910TR liquid scintillation counter (Packard, Meriden, CT, USA). The total protein content was measured by using a BCA protein assay kit and a FLUOStar Omega fluorescence microplate reader (BMG Labtech, Ortenberg, Germany). The protein assay for the OAT3 and mock cell lines was performed in four independent wells of each cell line, seeded and treated in the same manner as the cells used in the uptake assay. For all the OAT1, OAT3, and mock isotope uptake studies, the radiolabelled tracer accumulation (pmol) in each well was calculated by first determining the activity of the isotope (DPM∙pmol^−1^) from the radioactivity (DPM) of the donor sample divided by the theoretical isotope concentration (pmol), then dividing the total radioactivity in the well (DPM) to the specific activity of the isotope (DPM∙pmol^−1^).

### 2.7. Preincubation Assay

A range of preincubation solutions was prepared at the same concentrations of acamprosate applied in the inhibition assays. Acamprosate was dissolved in HBSS buffered with 10 mM HEPES and the pH was adjusted to 7.40 ± 0.01. The preincubation solutions were prewarmed to 37 °C and applied to the corresponding cells after removing the cell growth medium. The preincubation was done for 3 h, at 37 °C, without shaking. After the preincubation, the solutions were removed, and the inhibition experiment was performed as described in the Section 2.6.

### 2.8. Cell Viability Assay

The effect of the applied compounds on cell viability was measured in Lab. 2 by end-point toxicity assays using the Cell-Titer-Glo Luminescent cell viability assay following the assay instructions supplied by the provider (Promega Biotech AB, Nacka, Sweden). The cells were incubated for 3 h with increasing acamprosate concentrations (µM), 200 µM E3S or 1000 µM probenecid dissolved in uptake buffer. The incubation was done at 37 °C for the first 2.5 h and at room temperature for the final 0.5 h. The controls consisted of uptake buffer or SDS 0.5% (*w*/*v*). Since non-labelled E3S was dissolved in 0.5% methanol (*v*/*v*) before being applied to the cells, an extra condition was added where the incubation solution contained 0.5% methanol (*v*/*v*). For the 3-h incubation compounds showing less than 10% loss of viability as compared to control were considered non-toxic to the cells under this condition.

### 2.9. Acamprosate Intracellular Uptake and Inhibition of Uptake in HEK293-Flp-In OAT1, OAT3, and Mock Cells

The experiments were done on cells cultured in 24-well plates in Lab. 1. For the time-dependent uptake, the experiment was initiated by replacing the incubation buffer with 200 µL prewarmed 200 µM solution of acamprosate in uptake buffer. The plates were incubated in an orbital plate shaker at 37 °C and 100 rpm for 5, 15, 30, 45, 60, or 90 min and experiments were stopped by washing with 3 × 500 µL ice-cold buffer. For the dose-dependent uptake of acamprosate in OAT1 cells, the experiment was initiated by replacing the incubation buffer with 200 µL prewarmed donor solutions containing 0.33–33,000 µM acamprosate, while for the probenecid inhibition study the donor solutions contained 200 µM acamprosate and 0–1000 µM probenecid. The plates were incubated in an orbital plate shaker (Incubating microplate shaker from VWR International AB, Stockholm, Sweden) at 37 °C and 100 rpm for 10 min (time point at which substrate uptake was within the linear range) and experiments were stopped by washing with 3 × 500 µL∙well^−1^ ice-cold buffer. The cells were then lysed with 200 µL 50 nM warfarin in 60:40 acetonitrile (ACN):water (IS solution) and mixed thoroughly. The samples were moved to 96-well LC/MS-MS plates. The wells from the uptake experiment were rinsed again with 200 µL IS solution and the resulting volumes were added to the corresponding well on the same 96-well plate. Donor samples, diluted to reach a concentration in the dynamic linear range of the analysis method, zero calibration (IS solution), and blank samples (60:40 ACN:buffer and 60:40 ACN:water) were added on the same plate(s). The plates were covered with aluminum seals and centrifuged at 3500 rpm and 5 °C for 20 min and the amount of acamprosate in the supernatant was quantified by LC/MS-MS.

### 2.10. Analytical Procedure

Acamprosate was quantified in Lab. 1 using a Waters Xevo Triple-Quadrupole MS with electrospray ionization (Zspray) coupled to an Acquity UPLC (Waters, Milford, MA, USA). The system control was done with MassLynx software and the data analysis with TargetLynx software. The chromatographic separation was carried out on an Acquity UPLC HSS T3 column (Waters, Milford, MA, USA), 2.1 × 50 mm (1.8 µm) over a 2-min gradient with a flow rate of 0.500 mL/minute, at 60 °C, sample injection volume 10 µL. The autosampler temperature was 10 °C. Mobile phase A consisted of 5% acetonitrile and 0.1% formic acid in water and mobile phase B contained 0.1% formic acid in acetonitrile. The chromatographic run comprised of a flow of 95% mobile phase A and 5% mobile phase B for the first 0.5 min, followed by a linear increase of mobile phase B from 5% to 95% from 0.5 to 1.2 min, then a hold of 95% mobile phase B from 1.2 to 1.6 min, and finally a linear return to initial conditions from 1.6 to 2.0 min. The mass spectrometer was operated in negative ion mode. The ion spray temperature was 500 °C. The cone voltage was optimized to 20 V for IS and 38 V for acamprosate, while the collision energy was optimized to 24 V for both IS and acamprosate. Quantification was done by multiple reaction monitoring of parent and daughter ions. The mass transitions used were *m/z* 179.90 → 80.00 and 309.15 → 163.01 for acamprosate and IS, respectively, with a dwell time of 160 ms per transition. The validation of the analytical method is described in Appendix A.

### 2.11. Data Treatment

The obtained intracellular amounts (pmol) were normalized to the assay time (min) and cell surface area (cm^2^) or protein amount (mg protein) to obtain a term hereafter referred to as uptake rate. The uptake rate of acamprosate as a function of concentration was fitted to a Michaelis–Menten type equation which includes a saturable and a non-saturable uptake component,

(1) $$V=\frac{V_{max\;}\cdot \;\left[S\right]}{K_{m\;}+\left[S\right]}+P\;\cdot \;\left[S\right]\;$$

where *V* is the substrate uptake rate; *V_max_* is the maximal uptake rate; *P* is a constant related to non-saturable apparent uptake, i.e., factors such as passive diffusion, unspecific binding and residual compound present after washing steps, and [*S*] is the substrate concentration (µM). Uptake of [^14^C]-PAH, and [^3^H]-E3S as a function of concentration was fitted to Equation (2), and for acamprosate the non-saturable component (*P*∙[*S*]) was subtracted from each uptake rate and the data was refitted to a Michaelis–Menten equation which includes only a saturable component,

(2) $$V=\frac{V_{max\;}\cdot \;\left[S\right]}{K_{m\;}+\left[S\right]}$$

to obtain the Michaelis constant, *K_m_* (µM), i.e., the concentration at which the carrier achieves half the *V_max_*. The decrease in the carrier-mediated uptake rate in the presence of increasing concentrations of inhibitor was evaluated to determine the inhibitor concentration that causes 50% inhibition of transporter activity (IC_50_, µM). To calculate the IC_50_ of the substrate inhibition dose-response, data were fitted by non-linear least squares regression analysis and a one-site fit, according to the equation:

(3) $$V_{+i\;}=V_{+i,\;max}+\frac{\left(V_{-i}-V_{+i,\;max}\right)}{1+\frac{I^{n}}{IC_{50}^{n}}}$$

where *V*_+*i*_ is the uptake rate in the presence of the inhibitor (pmol∙cm^−2^∙min^−1^ or %); *V*_+*i, max*_ is the uptake rate at the maximum concentration of inhibitor (pmol∙cm^−2^∙min^−1^ or %); *V*_−*i*_ is the uptake rate in the absence of the inhibitor (pmol∙cm^−2^∙min^−1^ or %); [*I*] is the inhibitor concentration (µM) and *n* is the Hill Slope, fixed to −1. Assuming competitive interaction, equilibrium dissociation constants, *K_i_*, were calculated from the IC_50_ value for each substrate/inhibitor pair, at substrate *K_m_*, using the Cheng–Prusoff equation [24]. For concentration-dependent uptake of PAH in the presence of various acamprosate concentrations [I], the uptake rates were firstly fitted to Equation (2) using *K_m_*_,app_ and then to the following equation assuming competitive kinetics assuming a free fraction of 1 for [*I*] and [*S*]:

(4) $$V=\frac{V_{max\;}\cdot \;\left[S\right]}{K_{m\;}\left(1+\frac{\left[I\right]}{K_{i}}\right)+\left[S\right]}\;$$

The drug–drug interaction (DDI) index was calculated according to the equation:

(5) $$DDI\;index=\frac{{\left[I\right]}_{max,u}}{K_{i}}\;$$

where [*I*]*_max,u_* is the maximal unbound plasma concentration of the interacting drug, which can be defined as C_max,u_ the maximal observed plasma concentration (µM) of unbound inhibitor after oral administration or as C_0,u_ the initial plasma concentration (µM) after bolus intravenous administration. Input values for C_max,u_ or C_0,u_ calculated from clinical studies are listed in Appendix B, Table A1. According to FDA, an interacting compound with a DDI index ≥ 0.1 has the potential to inhibit the OAT1/3 carriers in vivo [25].

### 2.12. Statistical Analysis

The statistical analysis was performed using Graph Pad Prism, version 7.01. Differences among groups were determined using one-way analysis of variance (ANOVA) followed by Tukey’s multiple comparisons test. All tests were conducted using a confidence interval of 95% (*p ≤ 0.05). Data are reported as mean ± SEM from at least three independent cell passages unless otherwise stated.

## 3. Results

### 3.1. Establishment and Validation of HEK293-Flp-In OAT1 Transfected Cells

**Figure 2:**
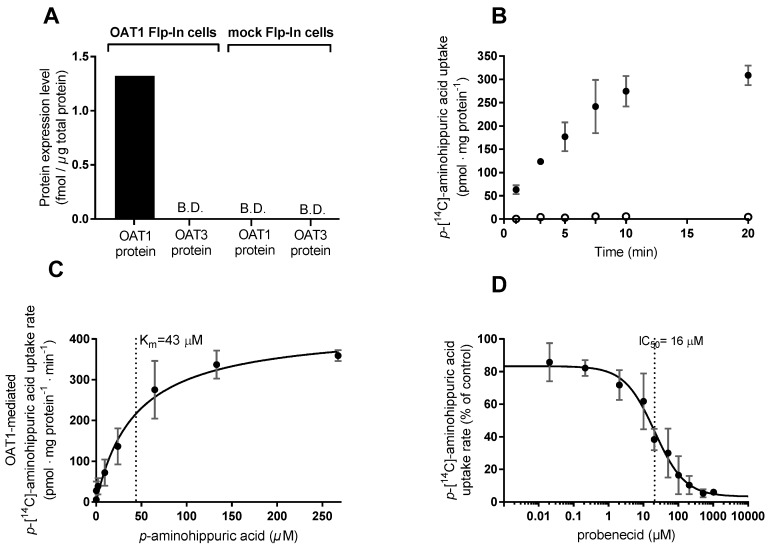
Functional characterization of HEK293-Flp-In cell lines stably transfected with *SLC22A6* (*OAT1*) or empty vector (mock-transfected). (**A**) OAT1 and OAT3 protein expression levels in HEK293-Flp-In OAT1 cells and HEK293-Flp-In mock cells. B.D., below the detection limit. (**B**) Time-dependent uptake of 9.5 µM *p*-[^14^C]-aminohippuric acid (PAH) in HEK293-Flp-In OAT1 expressing cells (closed circles) and HEK293-Flp-In mock cells (open circles). (**C**) The OAT1-mediated uptake rate of 0–277 µM of PAH using 0.02–9.5 µM [^14^C]-PAH in HEK293-Flp-In OAT1 cells (closed circles). (**D**) Effect of 0.02–1022 µM probenecid on the uptake rate of 9.5 µM [^14^C]-PAH in HEK293-Flp-In OAT1 cells. Each data point represents the mean ± SEM of replicates in three cell passages (**B**–**D**) or results from one cell passage (**A**) obtained at Lab. 1.

Stably transfected HEK293-Flp-In OAT1 cells and corresponding mock-transfected cells were successfully established in Lab.1, since proteomics data (Figure 2A) shows that the HEK293-Flp-In OAT1 cells express OAT1 protein (1.32 fmol/µg total protein) with no detectable OAT3 protein, whereas the Flp-In mock cells express neither OAT1 nor OAT3 protein at a detectable level.

The transport function of OAT1 in the stably transfected cells was investigated using PAH. The uptake of [^14^C]-PAH in the OAT1 expressing cells was relatively linear up to 7.5 min (Figure 2B). Subsequent uptake experiments were conducted for 5 min, where the uptake in the OAT1 cells was > 50-fold higher than in the mock cells. The concentration-dependent uptake rate of PAH (0.02–167 µM) followed Michaelis–Menten-like kinetics (Figure 2C). The kinetic parameters V_max_ and K_m,_ [^14^C]-PAH uptake are listed in Table 1.

**Figure A1:**
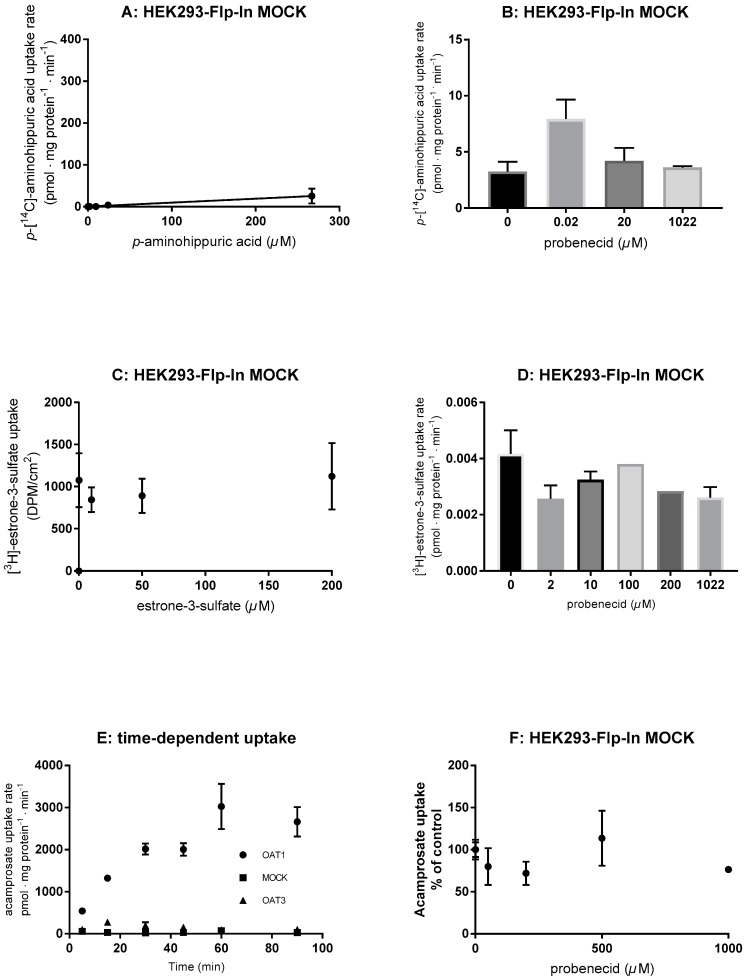
(**A**) The uptake rate of 0–277 µM of PAH using 0.02–9.5 µM [^14^C]-PAH in HEK293-Flp-In Mock cells. Each data point represents the mean ± SEM of replicates in three cell passages obtained at Lab. 1. (**B)** Effect of 0.02–1022 µM probenecid on the uptake rate of 9.5 µM [^14^C]-PAH in HEK293-Flp-In Mock cells. Each data point represents the mean ± SEM of replicates in three cell passages obtained at Lab. 1. (**C)** The uptake (DPM/cm^2^) 0.02 µM [^3^H]-E3S in HEK293-Flp-In Mock cells in the presence of 0–200 µM E3S. Each data point represents the mean ± SEM of replicates in three cell passages obtained at Lab. 2. (**D**) Effect of 0.02–1022 µM probenecid on the uptake rate of 0.02 µM [^3^H]-E3S in HEK293-Flp-In Mock cells. Each data point represents the mean ± SEM of replicates in three cell passages obtained at Lab. 2. (**E**) Time-dependent uptake of acamprosate in HEK293-Flp-In OAT1, Mock or OAT3 expressing cells. Each data point represents the mean±SD of three replicates in one cell passages obtained at Lab. 1. (**F**) Acamprosate uptake (% of control) with increasing concentrations of probenecid (0.02–1000 µM). The uptake of 200 µM acamprosate in HEK293-Flp-In Mock cells was measured in buffer pH 7.4. Each data point represents the mean ± SEM obtained in three cell passages obtained at Lab. 1.

The dose-dependent PAH uptake in the mock cells was not saturable at the same substrate concentrations (Figure A1A, Appendix B). Probenecid (0.02–1022 µM) inhibited the uptake of [^14^C]-PAH in a concentration-dependent manner in the HEK293-Flp-In OAT1 cells (Figure 2D) whereas no inhibitory effect was observed in the mock cells (Figure A1B). Probenecid IC_50_ and K_i_ for inhibition of [^14^C]-PAH uptake are listed in Table 2.

### 3.2. Establishment and Validation of HEK293-Flp-In OAT3 Transfected Cells

**Figure 3:**
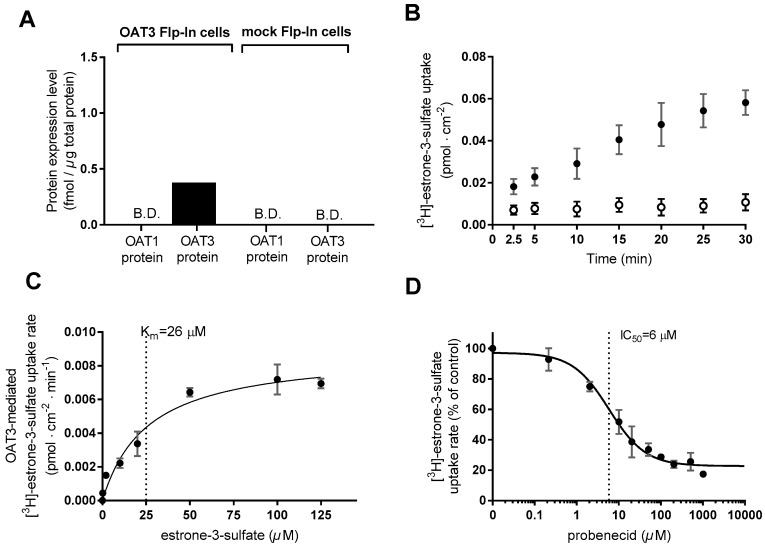
Functional characterization of HEK293-Flp-In cell lines stably transfected with *SLC22A8* (OAT3) or empty vector (mock-transfected). (**A**) OAT1 and OAT3 protein expression levels in HEK293-Flp-In OAT3 cells and HEK293-Flp-In mock cells. B.D., below the detection limit. (**B**) Time-dependent uptake of 0.02 µM (1 µCi/mL) [^3^H]-estrone-3-sulfate (E3S) in HEK293-Flp-In OAT3 expressing cells (closed circles) and HEK293-mock cells (open circles). (**C**) The OAT3-mediated uptake rate of 0–125 µM E3S spiked with 0.02 µM [^3^H]-E3S in HEK293-Flp-In OAT3 cells. (**D**) Effect of 0.02–1022 µM probenecid on the uptake rate of 0.02 µM [^3^H]-E3S in HEK293-Flp-In OAT3 cells (**D**). Each data point represents the mean ± SEM of replicates in three cell passages (**B**–**D**) obtained at Lab. 2 or results from one cell passage (**A**) obtained at Lab. 1.

HEK293-Flp-In cells stably transfected with *SLC22A8* and mock-transfected cells were established in Lab. 1. The proteomics result (Figure 3A) confirms that the HEK293-Flp-In OAT3 cells express OAT3 protein (0.38 fmol/µg total protein) with no detectable OAT1 expression. The Flp-In mock cells express neither OAT1 nor OAT3 protein to any detectable degree. The OAT3 transport function was determined in Lab. 2 using E3S as a substrate. [^3^H]-E3S showed a linear uptake in the OAT3 expressing cells for at least 20 min (Figure 3B). Subsequent uptake experiments were conducted for 20 min, where the substrate uptake in the OAT3 cells was > 5-fold higher compared to mock. The dose-dependent uptake rate of E3S (0.02–125 µM) was saturable with Michaelis–Menten-like kinetics (Figure 3C). V_max_, K_m_, and P of [^3^H]-E3S uptake are shown in Table 1. The dose-dependent E3S uptake in the mock cells was not saturable at the same applied concentrations of E3S (Figure A1C). Probenecid applied at concentrations of 0.2–1022 µM inhibited, in a concentration-dependent manner, the uptake of [^3^H]-E3S in the HEK293-Flp-In OAT3 cells (Figure 3D), whereas no inhibitory effect was observed in the mock cells (Figure A1D). Probenecid IC_50_ and K_i_ for inhibition of [^4^H]-E3S uptake are shown in Table 2.

### 3.3. Inhibition of OAT1 and OAT3 by Acamprosate and Standard Substrates

**Figure 4:**
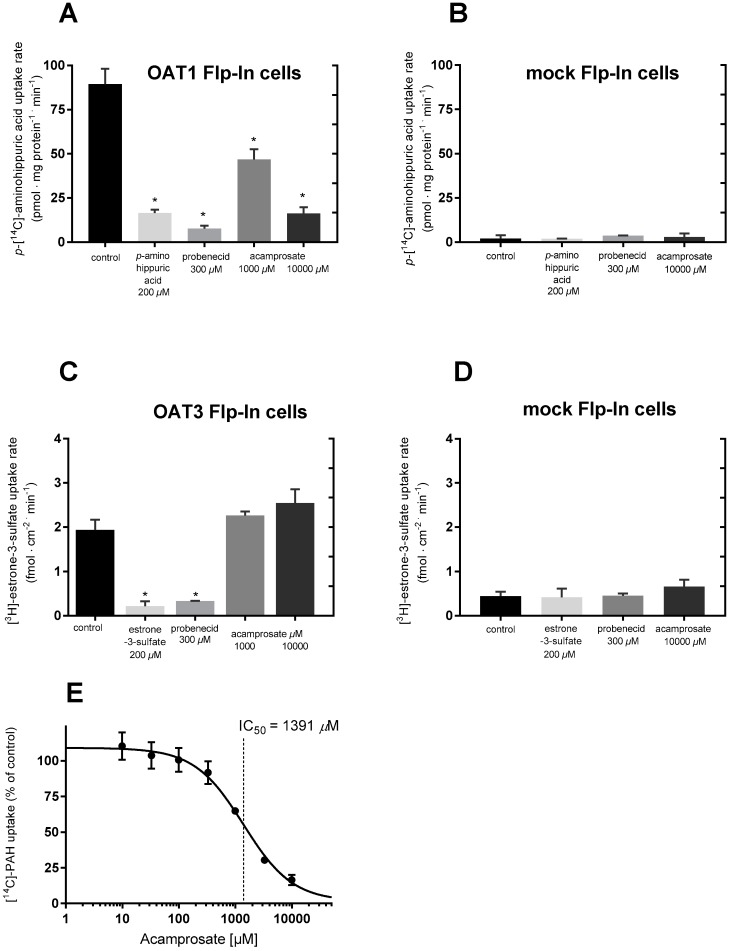
Evaluating the potential of acamprosate to inhibit substrate transport via OAT1/3 carriers. The uptake rate of *p*-[^14^C]-aminohippuric acid or [^3^H]-E3S in HEK293-Flp-In OAT1 or HEK293-Flp-In OAT3 expressing cells, respectively, compared with HEK293-Flp-In mock cells. The uptake rate of 9.5 µM (0.5 µCi/mL) [^14^C]-PAH was measured in the absence (control) or presence of 200 µM non-labelled *p*-aminohippuric acid, 300 µM probenecid or 1000 or 10,000 µM acamprosate in HEK293-Flp-In cells: (**A**) stably overexpressing the OAT1 carrier; (**B**) mock transfected. The uptake rate of 0.02 µM (1 µCi/mL) [^3^H]-estrone-3-sulfate in the absence (no inhibitor) or presence of 200 µM non-labelled estrone-3-sulfate, 300 µM probenecid or 1000 or 10,000 µM acamprosate into HEK293-Flp-In cells: (**C**) stably overexpressing the OAT3; (**D**) mock transfected cells (**E**): effect of 0–10,000 µM acamprosate on the uptake rate of 9.5 µM [^14^C]-PAH (% of control) in HEK293-Flp-In OAT1 expressing cells. The solid line is a fit of the data to Equation (3). Each bar depicts the mean ± SEM of replicates in three cell passages. (**A**,**B**,**E**) were obtained at Lab. 1, while (**C**,**D**) were obtained in Lab. 2. Differences as compared with the uptake rate in the control are indicated (* *p* ≤ 0.05).

The uptake of [^14^C]-PAH was measured in Lab. 1 in the presence of non-labelled PAH and probenecid in stably transfected HEK293-Flp-In OAT1 cells (Figure 4A) and the respective mock cells (Figure 4B). In the OAT1 cells, the uptake of [^14^C]-PAH without inhibitor was approximately 61-fold higher than in the Flp-In mock cell line. In OAT1 expressing cells, the uptake of [^14^C]-PAH was significantly decreased in the presence of 200 µM PAH and 300 µM probenecid (Figure 4A), whereas these had no effect on the uptake of PAH in mock cells (Figure 4B). Acamprosate also significantly decreased the uptake of PAH at both 1000 and 10,000 µM. This inhibition seemed to be concentration-dependent since 10,000 µM decreased the uptake more than 1000 µM, whereas acamprosate did not inhibit PAH uptake in mock-transfected cells.

The uptake of [^3^H]-E3S was measured in Lab. 2 in the presence of non-labelled E3S and probenecid in stably transfected HEK293-Flp-In OAT3 cells (Figure 4C). The uptake of [^3^H]-E3S was > 4-fold higher in the OAT3 cells compared to mock cells (Figure 4D). In OAT3 expressing cells, the uptake of [^3^H]-E3S was significantly decreased in the presence of non-labelled E3S (200 µM) and probenecid (300 µM), while no inhibition was observed in the mock cells. The 1000 or 10,000 µM acamprosate did not affect the uptake of [^3^H]-E3S in OAT3 expressing cells (Figure 4C) and 10,000 µM acamprosate did not affect uptake of [^3^H]-E3S in mock transfected cells (Figure 4D).

### 3.4. Evaluating If Acamprosate Is a Substrate for OAT1 and OAT3

**Figure 5:**
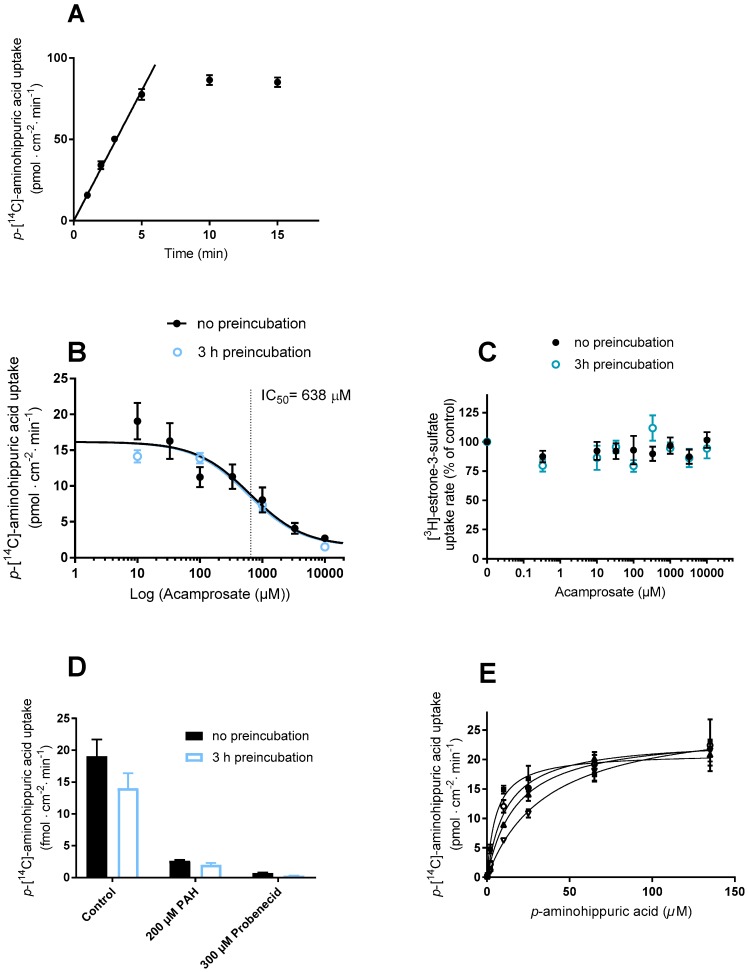
(**A**) Time-dependent uptake of 9.5 µM [^14^C]-PAH in HEK293-Flp-In OAT1 expressing cells. (**B**) Effect of 0–10,000 µM acamprosate on the uptake rate of 9.5 µM [^14^C]-PAH in HEK293- Flp-In cells with either applied concomitantly (black filled circles) or after 3 h preincubation with the same acamprosate concentration dissolved in cell medium (blue empty circles). (**C**) Uptake of 0.02 µM (1 µCi/mL) [^3^H]-estrone-3-sulfate (E3S) in HEK293-Flp-In OAT3 expressing cells with either applied concomitantly (black filled circles) or after 3 h preincubation with the same acamprosate concentration dissolved in HBSS buffered with 10 mM HEPES and the pH was adjusted to 7.40 ± 0.01 (blue empty circles). (**D**) Uptake of 9.5 µM [^14^C]-PAH in OAT1 expressing cells in the presence of 200 µM *p*-aminohippuric acid or 300 µM probenecid either applied concomitantly (black filled circles) or after 3 h preincubation with the indicated concentration dissolved in cell medium (open blue bars). (**E**) Concentration-dependent uptake of PAH in OAT1 HEK293-Flp-In cells in the presence of 0 µM (black filled squares), 625 µM (open squares), 1250 µM (open triangles), or 2500 µM acamprosate (open triangles). (**A**–**E**) The pH of the applied buffers was 7.4. Each data point represents mean ± SEM of replicates in three cell passages obtained in Lab. 2.

After observing the inhibition of OAT1-mediated transport in the presence of 1000 and 10,000 µM acamprosate in the HEK293-Flp-In OAT1 cells, the uptake of [^14^C]-PAH was investigated with increasing concentrations of acamprosate (0–10,000 µM) in Lab. 1 (Figure 4E) and Lab. 2 (Figure 5B), after measuring uptake of [^14^C]-PAH (Figure 5A). In Lab. 2 this uptake was linear for 5 min and an uptake time of 3 min was chosen. Acamprosate had a dose-dependent inhibition on [^14^C]-PAH uptake in OAT1 expressing cells with no apparent effect of a 3 h preincubation (Figure 5B). The estimated kinetic parameters IC_50_ and K_i_, as well as the DDI index, are listed in Table 2. A lack of inhibitory effect of acamprosate was observed for [^3^H]-E3S uptake for all applied concentrations, both with and without 3 h preincubation with the same acamprosate concentrations (Figure 5C). Similarly, a 3 h preincubation with PAH and probenecid did not significantly affect the uptake of [^14^C]-PAH (Figure 5D). To investigate the inhibitory mechanism of acamprosate on PAH uptake in OAT1 expressing cells, concentration-dependent uptake of PAH was measured in the presence of 0, 625, 1250, or 2500 µM acamprosate (Figure 5E).

Since the apparent K_m_-value for PAH increased while V_max_ remained constant, the ability of acamprosate to inhibit OAT1-mediated PAH transport is competitive. The kinetic parameters are given in Table 3.

**Figure 6:**
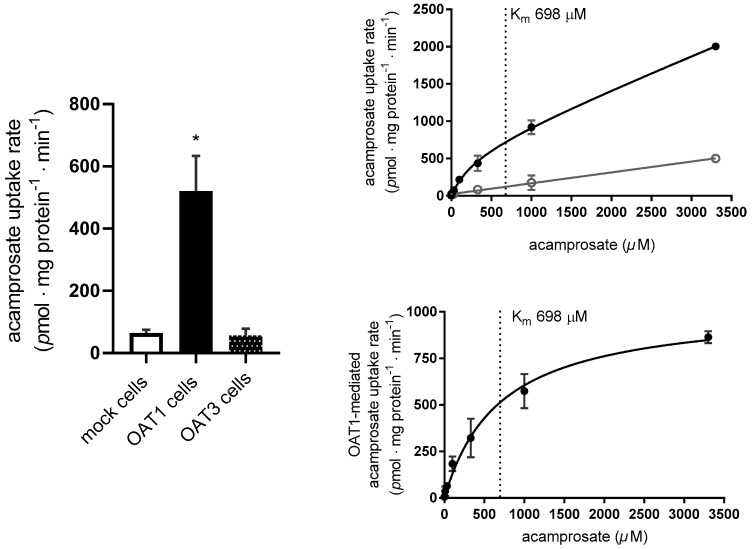
Evaluating acamprosate as a substrate for OAT1/3. (**A**) Uptake of 200 µM acamprosate measured for 10 min in HEK293-Flp-In mock, OAT1, and OAT3 cells. Differences as compared with the uptake rate in mock are indicated (* *p* ≤ 0.05). (**B**) The concentration-dependent uptake rate of 0.02*–*3300 µM acamprosate measured for 10 min in HEK293-Flp-In OAT1 cells (black closed circles) and Flp-In mock cells (grey open circles). (**C**) The OAT1-mediated uptake rate of 0.02*–*3300 µM acamprosate. Each data point represents the mean ± SEM obtained in three cell passages at Lab. 1.

To evaluate whether acamprosate is also a transported substrate of OAT1, the uptake of acamprosate at 200 µM was investigated in both the OAT1 and OAT3 expressing cell lines. A preliminary investigation showed that the uptake of acamprosate in OAT1 cells was relatively linear for at least 30 min (Figure A1E). Further uptake experiments were conducted for 10 min, where the uptake rate of acamprosate was approximately 20-fold higher than in mock-transfected cells. The uptake of acamprosate was statistically significantly higher in the OAT1 expressing cells compared to mock and OAT3 expressing cells where the uptake was similar (Figure 6A).

To further characterize the OAT1 mediated uptake of acamprosate dose-dependent acamprosate uptake studies were performed in the OAT1 expressing and mock cells. The concentration-dependent uptake rate of acamprosate (0.33–3300 µM) followed Michaelis–Menten-like kinetics (Figure 6B,C). The kinetic parameters V_max_, K_m_, and P of acamprosate uptake are listed in Table 1 and Table 3. The concentration-dependent uptake rate of acamprosate in the mock cells was not saturable for 0.33–3300 µM acamprosate (Figure 6B).

**Figure 7:**
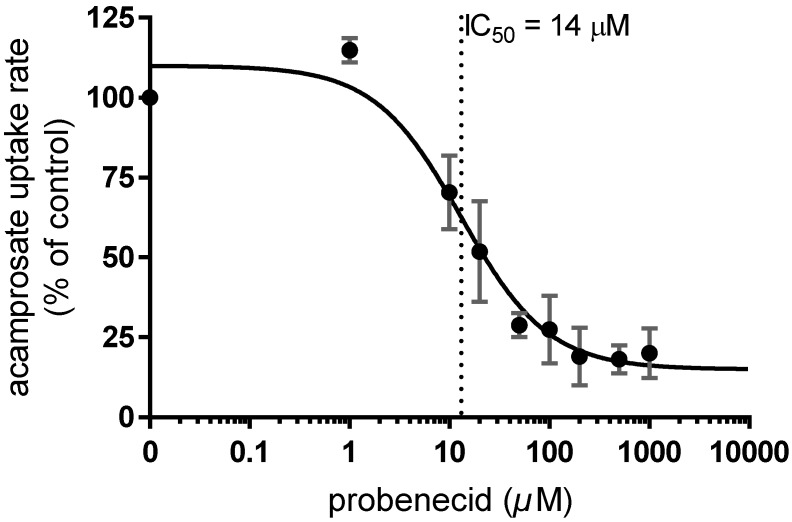
OAT1-mediated acamprosate uptake with increasing concentrations of probenecid (0.02–1022 µM). The uptake of 200 µM acamprosate in HEK293-Flp-In OAT1 cells was measured in buffer pH 7.4. Data are expressed as % of uptake rate (pmol∙mg protein^−1^∙min^−1^) of acamprosate without inhibitor (control). Each data point represents the mean ± SEM obtained in three cell passages obtained at Lab. 1.

Probenecid (0.02–1022 µM) inhibited the uptake rate of acamprosate in the HEK293-Flp-In OAT1 cells in a concentration-dependent manner (Figure 7), whereas no inhibitory effect could be observed in the mock cells (Figure A1F).

Probenecid IC_50_- and K_i-_values for inhibition of acamprosate uptake, together with probenecid DDI indexes with PAH and acamprosate are listed in Table 3.

### 3.5. Evaluation of Cell Toxicity upon Application of Substances

**Figure 8:**
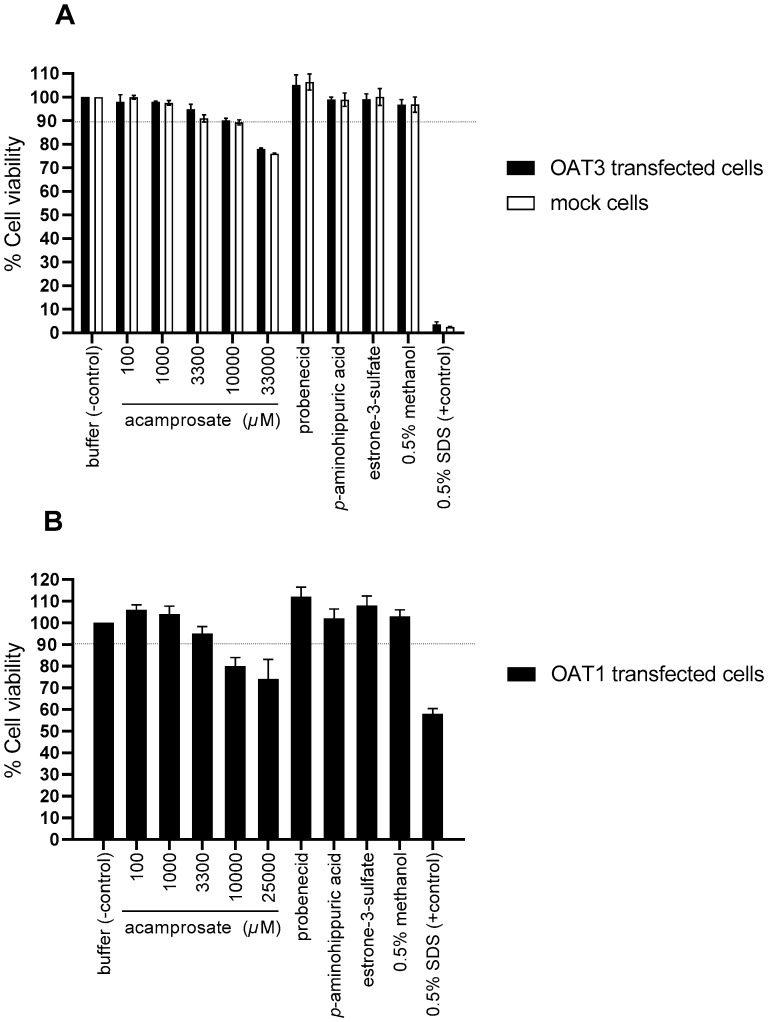
Evaluating the effect of the applied compounds on HEK 293 cell viability. The cell viability was assessed using CellTiter Glow and ATP was measured the cells were treated for 3 h with either uptake buffer (control) or 0.1, 1000, 3300, 10,000, 25,000, or 33,000 µM acamprosate, 1 mM probenecid, 1000 µM *p*-aminohippuric acid, 200 µM estrone-3-sulfate, 0.5% methanol, and 0.5% SDS. The measurement was done in OAT3 (**A**) or OAT1 (**B**) transfected cells (black bars) or mock cells (**A**, white bars). Each bar depicts the mean ± SEM of replicates in three (**A**) or four (**B**) independent cell passages obtained at Lab. 2.

To evaluate if acamprosate and the used standard compounds affected cell viability, a viability assay measuring cellular ATP was used. Figure 8 shows the measured % cell viability after a 3 h incubation. Incubating the cells with 0.5% SDS caused a decrease of more than 95% in cell viability in mock and OAT3 transfected cells, while in OAT1 transfected cells an approximately 50% decrease in viability was observed (Figure 8A,B). Incubating all three cell lines with up to 3300 µM acamprosate for 3 h did not cause a decrease in cell viability of more than 10%. Incubation with PAH, E3S, and 0.5% methanol did not decrease cell viability (Figure 8A,B). The cell toxicity assay used suggested that acamprosate concentration above 10 mM reduced cell viability in OAT3- and mock-transfected cells to below 90%. In OAT1 cells, slightly lower viability of approximately 80% was observed. The cell toxicity assay was performed after 3 h incubation to account for changes in the time-dependent inhibition studies, while affinity studies were also performed for 3 or 5 min, where the toxicity is likely lower. Therefore, data for 10 mM acamprosate was included for determining the affinity of acamprosate for OAT1 without preincubation.

## 4. Discussion

Organic anion solute carries such as OAT1 and OAT3 mediate the excretion of endogenous metabolites (e.g., uremic toxins, bile acids, bilirubin, fatty acids) and common drug substances (e.g., antibiotics, antivirals, diuretics, nonsteroidal anti-inflammatory drugs) and other xenobiotics, mainly organic anions, via basolateral uptake from the blood into the cytosol of the renal proximal tubule epithelial cells [26,27]. OAT1 and OAT3 are among the clinically relevant solute carriers recommended for evaluating potential DDIs for new investigational drugs [25]. Cells overexpressing transport proteins are the recommended system for investigating whether a drug substance can act as a potential in vitro inhibitor or substrate of a drug transporter [25]. In this work, HEK293-Flp-In cells overexpressing OAT1 or OAT3 solute carriers were developed and used to investigate the potential inhibitory effects of acamprosate on OAT1- and OAT3-mediated transport.

Acamprosate was, for the first time, shown to inhibit OAT1-mediated transport in OAT1 expressing cells. Data from the two different laboratories confirmed that acamprosate is inhibiting OAT1-mediated transport of PAH, with K_i_ values of 582–1229 µM. The differences in the obtained K_i_ values were not statistically different between the two laboratories. A recent study by Tatrai et al. [28] suggested that a quantitative potentiation of transporter inhibition by preincubation with the substrates could be observed for several classes of drug transporters, i.e., when the cells were preincubated with an inhibitor the measured IC_50_ value decreased. In our study, we also investigated if such an effect could be observed for the affinity of acamprosate for OAT1 but found that preincubation with acamprosate had no effect on the obtained affinity values. The interaction of acamprosate with OAT1-mediated transport of PAH was in the present study shown to be of a competitive nature.

In contrast, acamprosate did not inhibit the uptake of E3S via OAT3, at least at concentrations up to 10 mM. To investigate if preincubation with acamprosate resulted in a change in affinity, we preincubated the OAT3 expressing cells for 3 h with the same concentration of acamprosate used in the inhibition assay. Acamprosate still did not inhibit the uptake of E3S via OAT3. Tatrai et al. [28] showed that carriers such as OATP1B1, OATP1B3, OCT1, and OCT2 are more prone to preincubation effects, whereas such effects are lower for OAT3 and even lower for OAT1 and MATE carriers. Tatrai et al. suggested that substrates with a large molecular weight and a higher degree of lipophilicity are more prone to show preincubation effects. Acamprosate is a very hydrophilic compound (pK_a_ = 1.83, logP = −3.57) with a small MW (181.2 g/mol), and our limited data on a hydrophilic substance confirm that OAT1 and OAT3 show no preincubation effect on their affinity.

OAT1 and OAT3 have partially overlapping substrate specificity (e.g., PAH, probenecid) [29]. However, OAT3 has been shown to have reduced affinity for hydrophilic and small organic anions as compared to OAT1 [15]. The presence of OAT1 and OAT3 in the developed cell lines was functionally characterized by showing significant increase in uptake of OAT substrates, [^14^C]-PAH and [^3^H]-E3S, in OAT1 and OAT3 expressing cells, compared to mock-transfected cells. This demonstrated that both transfected cell lines were expressing functional OAT1 or OAT3 protein. An important element of the generation of robust cell lines is if they can be transferred between laboratories where cell handling procedures and standard culture and experimental procedures often differ. In the present study, some repetitive experiments were performed in two different laboratories. The time-dependent uptake of PAH and E3S were quite comparable with initial linear PAH uptake being 3–5 min and initial linear E3S uptake time being around 20 min. OAT1-mediated uptake of PAH could in both laboratories be inhibited almost completely with excess PAH and 300 µM probenecid. In Lab. 1, the K_m_ of PAH was estimated to 43 ± 13 µM in the transfected OAT1 cells, while a value of 7.7 ± 1.3 µM was found in Lab. 2. Although the values are different, both values are comparable to previously reported in vitro affinities obtained in other in vitro cell systems overexpressing human OAT1 (4–30 µM) [30,31,32,33,34,35,36,37,38] and with one PAH K_m_-value obtained in human kidney slices (≈39 µM) [15]. In addition, the IC_50_ of 16 µM obtained for the probenecid inhibition of PAH uptake is in good agreement with the published values (6–31 µM) from other cell models expressing human OAT1 [35,39,40]. For E3S uptake, the obtained K_m_ of 26 ± 6 µM in the OAT3-transfected cell lines is also comparable with the K_m_ values of 2–17 µM obtained cells overexpressing OAT1 [27,30,31,32,36,37] or with the E3S K_m_ obtained in human kidney slices (9–10 µM) [15].

The direct uptake of acamprosate in OAT1 and mock cells, showed that acamprosate uptake was significantly higher in the OAT1-transfected cells. This suggested that OAT1 recognizes acamprosate as a substrate and that acamprosate is a novel identified substrate for OAT1. The uptake of acamprosate in the OAT3 expressing cells was similar to the uptake in mock cells, suggesting that acamprosate is not transported via OAT3, consistent with the lack of affinity hereof. The uptake of acamprosate was saturable in the OAT1-expressing cells and non-saturable in the mock cells. The fitted non-saturable component (*P*∙[*S*]) brought a surprisingly high contribution to the total uptake rate. However, this component was similar to the total acamprosate uptake in the mock-transfected cells. This “passive” component can in fact be attributed to contributions from passive diffusion, unspecific binding to the cells or to compound still present in the residual fluid after repeated rinsing. However, it is unlikely that this component is predominantly due to passive transcellular uptake, as acamprosate is hydrophilic and >99% ionized at the assay pH, thus cannot permeate considerably via passive transcellular diffusion as shown earlier [18]. Nevertheless, after subtracting the uptake in OAT1 expressing cells with the apparent uptake in the mock cells, an OAT1-mediated saturable acamprosate transport was identified. The K_m_-value for the saturable acamprosate uptake was quite comparable to the estimated K_i_-value from the inhibition studies and was much higher than the K_m_-value obtained for PAH, which suggests that acamprosate is a lower affinity substrate than PAH. Other OAT1 substrates with K_m_-values similar to acamprosate are acyclovir, dimesna, methotrexate, and ganciclovir with K_m_-values of 342, 636, 554–724, and 896 µM [41,42,43], respectively. Acamprosate is, along with drugs such as adefovir, tenofovir, or ganciclovir, a relatively OAT1-specific substrate (due to uptake being at least 5× higher by OAT1 as compared to OAT3) [44]. However, the carrier specificity can only be assumed for these two solute carriers. Other organic anion carriers e.g., OAT2 and OAT4 have also shown to be expressed in the basolateral and apical membrane of the proximal tubule in the renal cortex, respectively [45]. These appear to be involved in the excretion of drugs in the kidney, such as acetylsalicylate (OAT2) or β-lactam antibiotics (OAT4) [46]. Even though these carriers have not been listed as clinically relevant by regulatory agencies [25], there is emerging research on their importance in the excretion of small anionic compounds. Thus, further studies to verify whether these other renal carriers might also contribute to acamprosate renal clearance may shed more light into the excretion mechanism of acamprosate.

Probenecid inhibited acamprosate uptake in OAT1 expressing cells with a K_i_ -value of 13 µM, a similar inhibition constant as the one obtained for probenecid inhibition of PAH in the same cell model. When acamprosate is administered intravenously in humans at doses ranging from 333 to 2130 mg its initial unbound plasma concentration (C_0,u_) reaches levels between 154 and 768 µM. Thus, it was relevant to investigate this interaction in more detail at the concentrations similar to the expected plasma levels of unbound drug, since there might be a risk of acamprosate being a perpetrator of OAT1-mediated transport when administered intravenously (unbound C_0,u_ = 154–768 µM for doses of 333–2130 mg) concomitant with other OAT1 substrates. However, when administered orally, acamprosate has been shown to reach plasma levels of around 1–9 µM in humans, which is approximately 100× lower than its OAT1 inhibiting K_i_-value at 638 µM. Thus, after oral administration, acamprosate may not reach high enough concentrations to act as a perpetrator drug for other compounds excreted via OAT1. Other drug substances with similar potency for inhibiting OAT1 are acetaminophen, acetylsalicylate, or cilastatin, with IC_50_ values of 639, 769, and 1470 µM [36,47], respectively. In rats, probenecid causes a 2.2-fold increase in acamprosate $AUC_{0}^{\infty }$ (area under the curve) and a decrease of unbound acamprosate CL_R_ to 0.45-fold, compared to control. However, it is worth noting that the expression of Oat1 is approximately 3-fold higher in rats compared to OAT1 in humans [48], thus perhaps this interaction might not occur to the same extent in humans. Nonetheless, probenecid is known to be involved in DDI with other OAT substrates with K_m_-values in the same range of acamprosate (e.g., acyclovir, OAT1 K_m_ 342 µM) in humans [49]. OAT mediated DDI by probenecid took to changes in the AUC of other inhibited drugs within 1.1–2.4-fold and their CL_R_ to 0.3–0.7-fold compared to control [50]. Thus, this DDI at the OAT1-level might constitute the molecular mechanism of the acamprosate-probenecid interaction found in rats [12] and might cause potential clinical DDI in humans with other OAT1 substrates when co-administered with acamprosate. Parvez et al. [30] reported an OAT1-mediated DDI index of 4.3 ± 0.64 for the interaction of probenecid with PAH, at an oral dose of 1 g probenecid. For oral doses of 0.5–2.0 g probenecid, we estimated for the probenecid–acamprosate interaction a DDI index ranging from 0.79 to 8.05, respectively, in a scenario where probenecid would be the perpetrator drug and acamprosate the victim drug. According to FDA, a DDI index ≥ 0.1 suggests a potentially clinically significant DDI that should be followed up by in vivo studies in humans [25]. Therefore, this newly described interaction may have DDI potential in humans. However, only clinical DDI studies in humans would be able to confirm the presence and extent of this interaction. Moreover, the calculation of the DDI index depends on the estimated K_i_-value, and as shown here the magnitude of this value can vary between different laboratories likely due to differences in experimental setups, causing a certain degree of variation to the DDI index. Therefore, this value should not be over-interpreted and treated with scientific scrutiny. Nevertheless, the kinetic parameters of acamprosate affinity for OAT1 presented in this study can help to identify the contribution of the OAT1 carrier to the in vivo clearance of acamprosate by in vitro-in vivo extrapolation (IVIVE). The K_m_ value of PAH to human kidney slices [15] reflects the K_m_ value obtained in our system and therefore it could be used as an input parameter for IVIVE. For increasing the accuracy of the IVIVE, additional work could be done to calculate a scaling factor from the absolute OAT1 expression data obtained in this study (1.32 fmol/µg total protein) to the OAT1 expression in the human renal cortex (5.33 ± 1.88 fmol/µg total membrane protein (CV 35.3%, *n* = 41 samples) [45].

## 5. Conclusions

In conclusion, we demonstrate that acamprosate is a substrate of OAT1, while it does not interact with OAT3. Thus, OAT1 may play an important role in active renal secretion of acamprosate in humans. An additional important finding is that probenecid, the prototypical OAT inhibitor, alters the OAT1-mediated uptake of acamprosate in a dose-dependent manner. Therefore, in analogy, the previously observed acamprosate–probenecid interaction in rats most likely occurs at the corresponding rat carrier (oat1, slc22a6) [12]. A clinically significant DDI index was calculated for the observed in vitro probenecid–acamprosate interaction. As the same interaction could occur between acamprosate and other OAT1 substrates or inhibitors, the cell lines can be used to investigate other acamprosate–drug, acamprosate–metabolite, or acamprosate–endogenous compound interactions. After acamprosate enters the cytosol of the renal epithelial cells, it uses another transporter for cellular efflux to the filtrate. Therefore, future studies should be aimed at identifying the transporter(s)/carrier(s) in the luminal membrane working in tandem with OAT1, which could be clinically relevant solute carriers or transporters such as OAT4, MATE1, P-gp, MRP2, MRP4, MATE2K, or BCRP.

## Figures and Tables

**Table 1:** Kinetic parameters associated with the uptake of *p*-aminohippuric acid and acamprosate, substrates for OAT1, and of estrone-3-sulfate, a substrate for OAT3, in HEK293-Flp-In OAT1 and OAT3 cells, respectively. Data are reported as mean ± SEM of replicates in three cell passages obtained in ^1^ Lab. 1 or ^2^ Lab. 2.

Substrate	*p*-Aminohippuric Acid ^2^	Acamprosate ^1^	Estrone-3-Sulfate ^2^
Carrier	OAT1	OAT1	OAT3
K_m_ (µM)	43 ± 13	698 ± 192	26 ± 6
V_max_ (pmol∙mg protein^−1^∙min^−1^)	429 ± 39	1028 ± 99	
V_max_ (fmol∙cm^−2^∙min^−1^)			8.8 ± 0.7
CL_T_ (cm/min)			5.6 × 10^−9^
CL_T_ (µL∙mg protein^−1^∙min^−1^)	10	1.5	
P (µL∙mg protein^−1^∙min^−1^)		0.35 ± 0.04	

V_max_ is the OAT1-mediated maximal uptake rate, K_m_ is the Michaelis constant (calculated only from the saturable uptake component); CL_T_ is the carrier-mediated clearance (V_max_/K_m_); P is the contribution of passive diffusion and potential effects from isotope dilution or non-specific cell binding.

**Table 2:** Kinetic parameters associated with the inhibition of uptake of *p*-aminohippuric acid and acamprosate via OAT1 and estrone-3-sulfate via OAT3 by acamprosate and probenecid in HEK293-Flp-In cells. Data are reported as mean ± SEM of replicates in three cell passages in ^1^ Lab. 1 or ^2^ Lab. 2.

Substrate			*p*-Aminohippuric Acid	Acamprosate	Estrone-3-Sulfate
Solute Carrier			OAT1	OAT1	OAT3
Inhibitor	probenecid	IC_50_ (µM) (log IC_50_ ± log S.E.M.)	16 ^1^ (1.20 ± 0.26)	14 ^1^ (1.14 ± 0.27)	6 ^2^ (0.77 ± 0.22)
		K_i_ (µM)	13 ^1^	11 ^1^	6 ^2^
		DDI index	0.67–6.81 ^a^	0.79–8.05 ^a^	1.69–17.23 ^a^
	acamprosate	IC_50_ (µM) (log IC_50_ ± log S.E.M.) IC_50_ (µM) (log IC_50_ ± log S.E.M.) IC_50_ (µM), 3 h (log IC_50_ ± log S.E.M.)	1391 ^1^ (3.1 ± 0.1) 638 ^2^ (2.8 ± 0.2) 582 ^2^ (2.8 ± 0.2)	-	>10,000 ^1,2^
		K_i_ (µM) K_i_ (µM) K_i_ (µM)	1140 ^1^ 315 ^2^ 287 ^2^	-	-
		DDI index	0.001–0.008 ^a^ 0.14–0.70 ^b^	-	<<0.02 ^a, b^

^a^ oral administration; ^b^ intravenous administration; IC_50_ is the inhibition constant and K_i_ is the equilibrium dissociation constant if assuming competitive inhibition. DDI index represents the calculated drug–drug interaction index. The inputs for the dose, C_max_, C_max,u_, f_u_ used for calculating the DDI indexes are listed in Table A1 in Appendix B.

**Table 3:** Kinetic parameters associated with the uptake of *p*-aminohippuric acid (PAH) in the presence of various acamprosate concentrations in OAT1 expressing HEK293 cells. Data are reported as mean ± SEM of replicates in three cell passages obtained at Lab. 2.

Substrate—PAH	0 (µM) Acamprosate	625 (µM) Acamprosate	1250 (µM) Acamprosate	25,000 (µM) Acamprosate
K_m, app_ (µM)	5.6 ± 1.4	11.9 ± 3.2	17.9 ± 3.2	36.5 ± 7.4 ^*^
V_max, app_ (pmol∙cm^−2^∙min^−1^)	21.1 ± 1.1	23.4 ± 1.7^ns^	24.4 ± 1.3^ns^	27.6 ± 2.1^ns^
K_m_ (µM)	7.7 ± 1.3			
V_max_ (pmol∙cm^−2^∙min^−1^)	23.2 ± 0.7			
K_i_ (µM)	1229 ± 341			

K_m_ and V_max_ values were compared using an ANOVA test, where means were significantly different for K_m_ values and not significantly different for V_max_ values. Values were compared to 0 (µM) acamprosate using a Dunnett’s post-test; * *p* < 0.05 and ns: not significant.

## Appendix A

### LC/MS-MS Method Validation

The method validation was done according to the U.S. Food and Drug Administration (FDA) Bioanalytical Method Validation Guidance for industry [51]. The validated parameters were selectivity, sensitivity, linearity, accuracy, precision, and dilution integrity. *Selectivity*. Blank cell matrix samples showed a response of 17.35% ± 3.46% (mean ± S.D., *n* = 4) of the lower limit of quantification (LLOQ) at the retention time of the acamprosate, therefore, we can assume the components of the cell matrix do not interfere with the detection of acamprosate. The transport buffer blank and zero calibration blank (containing only the internal standard warfarin) had a signal <20% of LLOQ. We did not evaluate interference from metabolites as acamprosate is not metabolized in enterocytes. *Sensitivity*. LLOQ for acamprosate was 3.16 nM (0.57 ng/mL). This amount was quantified with an accuracy of 16.06% from nominal concentration and a precision of 10.78% (*n* = 5 replicates). *Linearity*. The triplicate standard curve contained nine concentration levels and was linear for the range of 3.16 nM to 10.00 µM (0.57–1802 ng/mL), R^2^ = 0.9968. The back-calculated average concentrations of all non-zero calibrators were within 85%–105% of their nominal concentrations except LLOQ who was between 80% and 120% of its nominal concentration. *Accuracy and precision*. The accuracy and precision of the method was controlled by measuring QC samples prepared independently from the calibration standards, with a final concentration of 3.16 (LLOQ), 60.00 (low-range QC), 600.00 (mid-range QC), and 6000.00 (high range QC) nM acamprosate, in five replicates and measured in at least three independent runs. The intra-run accuracy for low-, mid-, and high-range QC samples was between 2.02% and 8.84 % while the precision was 4.15% and 8.88%. The inter-run measurements had an accuracy of 6.12% and 11.33% and a precision of 6.90% and 11.59%. *Dilution integrity*. The 200.00 µM standards diluted 1/100 gave back-calculated concentrations in the range of 85%–115% of their nominal concentration, and a CV of 5.93%.

## Appendix B

Calculation of drug–drug interaction (DDI) indexes. The calculation of DDI indexes was performed according to the 2020 FDA guidance for evaluating in vitro drug–drug interaction [25] and as described in the Method section of the main manuscript. Table A1 lists the unbound maximal concentration (C_max_) and fraction unbound (f_u_) values collected from clinical studies of acamprosate and probenecid, the IC_50_ values obtained in the present study, and for acamprosate given as the interval for obtained values, and the calculated unbound maximal concentrations C_max,u_ and DDI indexes. Figure A1: Control experiments in mock-transfected cells and time-dependent uptake of acamprosate.

**Table A1:** Maximal mean (±SD) plasma concentration (C_max_), fraction unbound in plasma (f_u_), and in vitro obtained IC_50_ values together with the calculated unbound maximal plasma concentration (C_max,u_) and DDI indexes for probenecid–PAH, probenecid–acamprosate, probenecid–E3S and acamprosate–PAH interactions. N.A. not applicable.

Inhibitor	Adm. Route	Dose	C_max_	f_u_	C_max,u_	*p*-Aminohippuric Acid (PAH)		Acamprosate		Estrone-3-Sulfate	
						IC_50_	DDI Index	IC_50_	DDI Index	IC_50_	DDI Index
		mg	µM		µM	µM		µM		µM	
Probenecid	Oral	500	124 ± 30 [52]	0.07 [53]	8.66 ± 2.09	13	0.67 *	11	0.79 *	6	1.69 *
		1000	244 ± 94 [52]	0.11 [53]	26.83 ± 10.37		2.06 *		2.44 *		5.22 *
		2000	521 ± 166 [52]	0.17 [53]	88.53 ± 28.24		6.81 *		8.05 *		17.23 *
Acamprosate	IV bolus	333	153 ± 38 [7]	1 [5]	153 ± 38	287–1229	0.12–0.53 *	-		>>10,000	N.A.
		666	214 ± 10 [6]		214 ± 10		0.17–0.75 *				
		710	230 ± 64 [10]		230 ± 64		0.19–0.80 *				
		1420	486 ± 106 [10]		486 ± 106		0.40–1.69 *				
		2130	768 ± 149 [10]		768 ± 149		0.62–2.68 *				
	Oral solution	333	1.8 ± 0.8 [9]		1.8 ± 0.8		0.00–0.01				
		666	4.3 ± 1.4 [9]		4.3 ± 1.4		0.00–0.01				
		1332	5.0 ± 1.7 [9]		5.0 ± 1.7		0.00–0.02				
		2664	8.6 ± 4.1 [9]		8.6 ± 4.1		0.01–0.03				
	Oral tablet	666	1.4 ± 1.1 [6,54]		1.4 ± 1.1		0.00–0.00				
		1332	1.5 ± 0.1 [55]		1.5 ± 0.1		0.001–0.01				

* marked DDI index with values ≥0.1, ergo clinically significant, according to the FDA guideline [25].
